# Evaluation of Exercise Mobile Applications for Adults with Cardiovascular Disease Risk Factors

**DOI:** 10.3390/jcdd10120477

**Published:** 2023-11-28

**Authors:** Shiqi Chen, Yin Wu, Erica L. Bushey, Linda S. Pescatello

**Affiliations:** 1 Department of Kinesiology, University of Connecticut, Storrs, CT 06269, USA; yin.wu@hhchealth.org (Y.W.); erica.bushey@uconn.edu (E.L.B.); linda.pescatello@uconn.edu (L.S.P.); 2 Institute for Collaboration on Health Intervention and Policy, University of Connecticut, Storrs, CT 06269, USA; 3 Department of Research, Hartford Hospital, Hartford, CT 06102, USA

**Keywords:** exercise prescription, systematic review, digital health

## Abstract

Objective: To conduct a systematic review to determine if there are exercise mobile applications (apps) that can produce evidence-based, individualized exercise plans. Materials and Methods: We searched the Apple Store and Google Play for exercise apps with terms related to exercise and health. Exercise apps were eligible if they: (1) had a ≥4 out of 5 overall rating with ≥1000 reviews; (2) were free to download; and (3) were not gender specific. Exercise apps were evaluated via the evidence-based exercise prescription (ExRx) standards of the American College of Sports Medicine (ACSM) and American Heart Association. For the exercise app evaluation criteria, an app was included if it (1) was evidence-based; (2) contained a preparticipation health screening protocol; (3) built a cardiovascular disease (CVD) risk factor profile; (4) prioritized one chronic disease or health condition to focus on; (5) framed the exercise plan by the frequency, intensity, time, and type principle (FITT) of ExRx; and (6) specified special considerations. Results: Of the 531 potentially qualifying apps, 219 qualified. The qualifying apps were rarely evidenced-based (0.5%) or had a preparticipation screening protocol (3.7%). Only 27.7% built CVD risk factor profiles. Most apps (64.8%) focused on body image and/or athletic performance. Only 4.3% focused on chronic diseases or health conditions, while the remainder (34.5%) did not disclose a focus. No app framed the exercise plans by the FITT of ExRx. Only 1.4% of the apps specified special considerations. Conclusion: There are no commercially available exercise apps that generate an evidence-based, individualized exercise plan for adults with CVD risk factors.

## 1. Introduction

Of the 258 million people in the United States (US), 65 million or 25% are physically inactive [1]. Nearly 50% of US adults live with one or more CVD risk factors including physical inactivity [2]. In addition, physical inactivity clusters with other CVD risk factors, including obesity, type 2 diabetes mellitus, dyslipidemia, and hypertension [3]. CVD is the leading cause of death contributing to one in three deaths in the US and across the world [4]. Exercise is regarded as one of the most effective lifestyle interventions to prevent and treat CVD and its risk factors [5,6]. Accordingly, the American Heart Association (AHA), American College of Cardiology [2,7], American College of Sports Medicine (ACSM) [8], and World Health Organization [9], among other professional organizations throughout the world, recommend exercise as a key first-line lifestyle approach to prevent and treat CVD.

Despite the many health benefits of leading a physically active lifestyle, only 30% of primary care physicians recommend exercise to their patients [10,11] even though a clinician’s recommendation to exercise is a strong incentive for patients to exercise [12]. The reasons for this concerning statistic are that clinicians do not have the guidance, training, confidence, and/or time to counsel patients to exercise [6,7,9,11,12]. Mobile exercise applications (apps) offer a promising solution for this problem. Exercise mobile apps have proliferated because of their low consumer cost, high population reach, and immediate accessibility. There are over 100,000 exercise apps in the Apple App Store, and 165,000 when including Android’s Google Play [13,14]. Modest evidence indicates app-based interventions are effective in improving physical activity [15,16].

An exercise prescription (ExRx) is the process whereby an individualized physical activity program is structured by *F*requency (How Often?), *I*ntensity (How Hard?), *T*ime (How Long?), and *T*ype (What Kind?) of exercise or FITT [15,16]. In the context of health, ExRxs are most effective if they target specific health outcomes [7,8,10,12,17,18]. As of this review, it is still unknown whether there are publicly available exercise apps that clinicians can use to design personalized ExRxs for adults with CVD risk factors. We conducted this systematic review to evaluate commercially available exercise apps to determine if evidence-based exercise apps exist on the market that clinicians can use to prescribe individualized ExRx for adults with CVD risk factors and other chronic diseases and health conditions.

## 2. Methods

### 2.1. Search Strategy and Methods

This systematic review is registered at PROSPERO (CRD42021291240) and was conducted in accordance with the Preferred Reporting Items for Systematic Reviews and Meta-analyses (PRISMA) Statement [19]. There are over 100,000 exercise apps in the Apple App Store, and a total of 165,000 when including the Google Play App Store [15,16]. Consistent with the protocols of Breton et al. [20] and Azar et al. [21], we reviewed the top-ranked exercise apps on the Apple Store and Google Play with the highest outreach potential. Accordingly, the included exercise apps met the following inclusion criteria: (1) had ≥1000 user reviews; (2) had a ≥ four out of five star rating based on the users’ reviews; (3) were free to download, although they could still contain content to purchase; and (4) were not gender specific [20,21].

**Figure 1:**
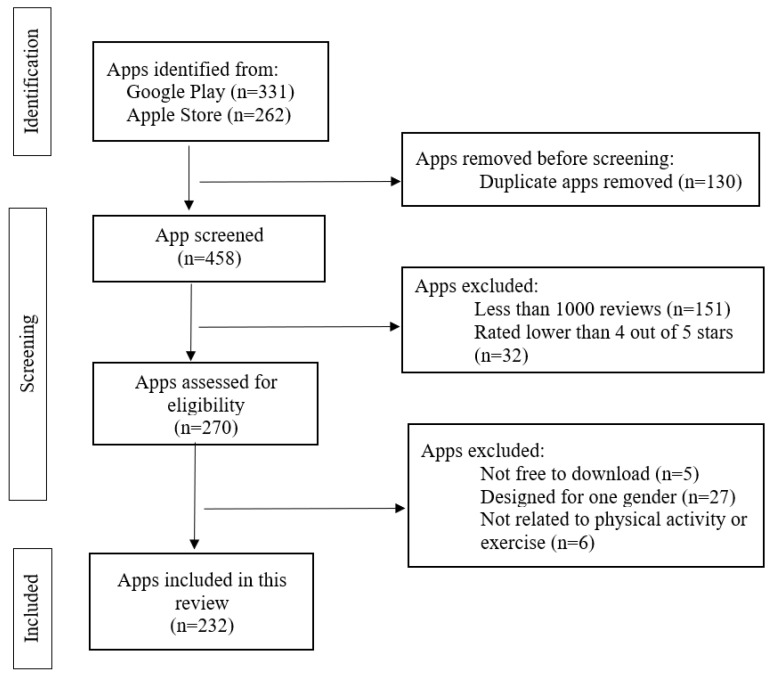
PRISMA diagram of the Search Methods and Strategy.

After consulting with a medical librarian (JL), and staying consistent with the strategies of Gustavo et al. [22] and Brianna et al. [23], we systematically searched for smartphone apps on the Apple Store and Google Play with the search terms “exercise for health”. The search was performed on 10 November 2023. The search revealed 593 potentially qualifying exercise apps. Trained coders (EB, YW, RS, and SC) screened the exercise apps from the information displayed on the app downloading webpage including name, introduction, screenshots of the app interfaces, and price. Each exercise app was screened by two coders independently using a standardized data extraction form. If an exercise app was available on both the Apple Store and Google Play and had the same content, only apps on Google Play were reviewed. After removing 323 duplicate apps, 270 potentially qualifying apps were downloaded for further review. In the end, 232 exercise apps qualified. See Figure 1 for the Preferred Reporting Items for Systematic Reviews and Meta-Analyses (PRISMA) diagram of the search strategy and methods.

### 2.2. Data Extraction and Coding

We reviewed each exercise app with seven questions based on the ExRx standards set forth by the ACSM and AHA [24,25] after consulting with experts in the field of ExRx that had an important role in constructing these standards. Table 1 presents the seven questions upon which each exercise app was evaluated. Question One asked if the app specified the scientific foundation upon which the app was based (e.g., professional organization guidelines such as from the ACSM and AHA). Question Two asked if the exercise app included an exercise preparticipation health screening function for users [24,26]. Question Three asked if the exercise app built a CVD risk factor profile for users including the major CVD risk factors of hypertension, diabetes mellitus, dyslipidemia, obesity, and physical inactivity [24,25]. Question Four asked if the app focused on one or more of the user’s health conditions or body image or athletic performance [24]. Question Five asked if the exercise app provided recommendations for all four types of exercise (i.e., aerobic, resistance, neuromotor, and flexibility) [18,24]. Question Six asked if the app provided the Frequency, Intensity and Time (FIT) information for each type of exercise recommended [24]. Last, Question Seven asked if the exercise app provided special considerations to ensure safety (e.g., individuals with diabetes who take insulin or medications that increase insulin secretion should monitor blood glucose before and after exercise to avoid hypoglycemia.) and maximize the health benefits (e.g., a reduction in body weight, if achieved simultaneously with exercise, will generate additional health benefits among individuals with obesity compared to exercise alone or weight loss alone) [24].

### 2.3. Statistical Analysis

Descriptive statistics of the qualifying apps were obtained using Microsoft Excel for Microsoft 365 MSO (Version 2205). We counted the number of apps that met the criteria for each app function (e.g., we reported 10 apps that met the criteria for function B of Question 1), and calculated the percentage (e.g., [10 apps for function B for Question 1/the total number of apps = 232] × 100% = 4.3%).

## 3. Results

The initial search yielded 593 potential qualifying apps. After triaging, 232 apps qualified. Among these apps, 182 were from Google Play and 130 were from the Apple Store. See Figure 1 for the searching and triaging process.

### 3.1. The Scientific Evidence Base

Of the qualifying apps, only one (0.4%) app, the *7 Minute Workout App—Lose Weight in 30 Days!* [27] app, stated that the exercise plan was based upon scientific evidence. This app creates a daily 7 min exercise plan for users. The app allows users to set exercise goals, including body image and weight control. The developers of this app stated that the effectiveness of their 7 min workouts was supported by scientific studies; however, the studies were not referenced [27]. The remainder (*n* = 218, 99.6%) of the qualifying apps provided no information regarding whether the app was based upon scientific evidence or not. Meanwhile, the developers of nine (4.3%) apps reported their exercise plans were designed by expert(s) or professional(s). However, the credentials or affiliations of these expert(s) or professional(s) were only disclosed in one (11.1%) of these nine apps. This app, the *Jillian Michaels | The Fitness App* [28] involved a certified trainer from the National Exercise & Sports Trainers Association. The National Exercise & Sports Trainers Association is an organization that offers certification courses for exercise professionals such as personal trainers and physical therapists [29]. The remainder of these apps (*n* = 8, 88.8%) used unspecified terms to describe the credentials of experts or professionals, such as *certified trainer* or *yoga instructor*.

### 3.2. The Preparticipation Health Screening Protocol

An essential first step in constructing an exercise plan is performing an exercise preparticipation health screening of which the ACSM’s protocol is considered a standard in the field [24,30]. Surprisingly, none of the qualifying apps included an exercise preparticipation health screening function. About a third (*n* = 80; 34.5%) of the qualifying apps recommended users to consult with professionals regarding the safety to start exercise in the legal disclaimers of the app. However, only a third (*n* = 26; 32.5%) of these seventy-nine apps required users to read the legal disclaimers; while the remainder (*n* = 54; 67.5%) presented legal disclaimers at places where the users did not have to read them before using the apps. Over half (*n* = 152; 65.9%) of the qualifying apps did not have any relevant information regarding an exercise preparticipation health screening.

### 3.3. The Cardiovascular Disease Risk Factor Profile

The second step of designing an exercise plan for adults with CVD risk factors is to identify the CVD risk factors [24,30]. Only one (0.4%) of the qualifying apps, Fitpaa *💪 Weight Loss/Gain, Bodybuilding, Six Pack* [31], had this function. This app creates an exercise plan for users according to the goals of exercise chosen by the user. Examples of the goals of exercise that can be chosen in this app include body image, fitness, diabetes, dyslipidemia, and hypertension control. This app had a comprehensive CVD risk factor profile function where users enter their BMI, age, blood glucose, blood pressure, blood lipids–lipoproteins, and physical activity level. This app categorized baseline physical activity levels as follows: beginner (workout less than 30 min per week), intermediate (workout 0.5–3 h per week), and advanced (workout 3–5 h per week). About a third (*n* = 90, 38.8%) of the qualifying apps collected information on some but not all CVD risk factors (see Table 1). The remainder (*n* = 142; 61.2%) of the qualifying apps collected no information on CVD risk factors.

### 3.4. Prioritization of the Chronic Diseases or Health Conditions

The third step of planning an exercise plan is prioritizing the CVD risk factor or chronic disease or health condition that the FITT ExRx will focus on [24,30]. Only one (0.4%) of the qualifying apps, *Fitpaa 💪 Weight Loss*/*Gain, Bodybuilding, Six Pack* [31], had this function. Customer service staff from this app contacts each user and discusses the focus of the exercise plan related to their chronic diseases or health conditions. Due to privacy concerns, we did not let the service staff of the app contact us when we were reviewing the app. No other information was provided on how the service staff would discuss the focus of the exercise plan with users regarding the prioritized chronic disease or health condition. Meanwhile, eight (3.7%) of the qualifying apps were designed to target one specific health condition. Of these eight apps, six (75.5%) targeted back pain, one (12.5%) targeted neck pain, and one (12.5%) targeted musculoskeletal discomfort. Of note, most (*n* = 155; 66.8%) of the qualifying apps did not focus on any chronic disease or health condition; rather, they focused on improving body image (e.g., gaining muscle) and/or athletic performance (e.g., increasing running speed). The remaining (*n* = 69; 29.7%) apps did not disclose any chronic disease or health condition to focus their exercise plans.

### 3.5. Framing the Exercise Plans by the FITT ExRx

The fourth step in designing an exercise plan is to frame the exercise plan by the ACSM FITT principle of ExRx [24,30]. No app framed the exercise plan according to the FITT principle of ExRx. Among the qualifying apps, 42 (18.1%) detailed the frequency, intensity, and time (FIT) in the exercise plans. About half of the qualifying apps (*n* = 116, 50.0%) provided some but not all of the FIT information. Of note, since these 158 apps did not design exercise plans for any specific chronic disease or health condition, they provided no clear rationale for why a specific FIT was determined. Last, 74 (31.9%) of the qualifying apps did not provide any FIT information. For example, one app suggested users run more than 8 miles per week without specifying pace or time [32].

Only 10 (4.3%) of the qualifying apps recommended the four types of exercise—aerobic, resistance, neuromotor, and flexibility—as per the ACSM guidelines [17]. Most (*n* = 164; 70.7%) of the qualifying apps recommended only aerobic and/or resistance exercise. Nearly half (*n* = 49; 21.1%) of the qualifying apps did not recommend the specific types of exercise; rather, the exercise plan consisted of providing exercise examples and the users then chose the exercises they preferred to perform. For example, one app provided various exercise options such as Yoga, body-weight resistance training, dancing, among others, without providing information on which type of exercise these exercises were [33]. Of note, we did not review the types of exercise recommended in nine (4.1%) of the qualifying apps since payment was required.

### 3.6. Special Considerations

The fifth and last step in designing an exercise plan is listing the special considerations associated with the CVD risk factor, chronic disease, or health condition the exercise plan targets [3,4]. None of the included apps provided a list of special considerations associated with the targeted CVD risk factor, chronic disease, or health condition as specified by ACSM [17,18,24,30]. For example, when targeting obesity, special considerations for ExRx may include: (1) utilization of goal setting to target short- and long-term weight loss, including a target of a minimal reduction in body weight of at least 3–10% of initial body weight over 3–6 months; (2) target reducing current energy intake to achieve weight loss. This reduced energy intake should be combined with a reduction in dietary fat intake; and (3) weight loss beyond 5–10% may require more aggressive nutrition, exercise, and behavioral intervention, among others [17,18,24,30]. Only three (1.3%) of the qualifying apps included some information related to special considerations. For example, *Back pain exercises at home* [34] is an app that provides a video demonstration of resistance and stretching exercises targeting back muscles. The app encourages users to discuss special considerations with a professional to optimize the health benefits and minimize the risks of exercise. Nearly all (*n* = 229; 98.7%) of the qualifying apps did not include any content related to special considerations.

## 4. Discussion

This systematic review investigated if there were publicly available exercise apps that can help clinicians prescribe exercise to patients with CVD risk factor(s), as currently most clinicians are not routinely prescribing exercise due to a lack of guidance, training, confidence, and/or time to counsel patients to exercise [6,7,9,11,12]. Unexpectedly, we did not find a single app clinicians could use for patients with CVD risk factors to generate FITT ExRx that met the evidence-based professional standards of ACSM and AHA [2,24]. Indeed, only 1 exercise app of the 232 qualifying apps provided scientific evidence to support the foundation and delivery of their exercise programs (Table 1 Question 1). Moreover, two thirds (~67%) of the exercise apps we evaluated did not meet any of the seven review criteria in Table 1.

The second review criterion was if the app had a function to perform exercise preparticipation health screening to determine whether medical clearance was needed. Preparticipation exercise health screening should be performed because it speaks to the safety of exercise by reducing the likelihood of adverse events that can result from exercise [18,26,35]. In this review, no app included an exercise preparticipation health screening function nor did they require medical clearance for the users to access the exercise content (Table 1 Question 2). About one third of the apps (~36%) had a legal disclaimer recommending the user consult a professional prior to exercising. For example, in the legal disclaimer of the app named “*FitOn—Free Fitness Workouts & Personalized Plans*” [36], it stated users should consult with a healthcare provider before starting to exercise for any health and safety concern [18,26,35].

The third review criterion by which we evaluated the qualifying exercise apps was if the app built a CVD risk factor profile that includes the major CVD risk factors of hypertension, diabetes mellitus, dyslipidemia, obesity, and physical inactivity [4,25,37,38,39]. Just over one third of the apps we reviewed collected information on at least one of the major CVD risk factors (Table 1 Question 3). Only one app built a comprehensive CVD risk factor profile for users. The fourth review criterion by which we evaluated the qualifying exercise apps was if the app systematically identified and prioritized the risk imposed by the CVD risk factors when generating FITT ExRx to optimize CVD prevention and management [25,35,40,41]. An interesting finding that may explain why CVD risk factors were not routinely assessed in the apps was that very few (~4%) of the apps were designed for the purpose of health improvement; meanwhile, while nearly two thirds of the apps (~65%) focused on fitness (such as strength improvement) or body image (such as “having six abs”) (Table 1 Question 4). Even among the apps that focused on health rather than fitness, just one collected information on a CVD risk factor profile only if the goal of exercise was health-related [31]. This app, “*Fitpaa 💪 Weight Loss/Gain, Bodybuilding, Six Pack*”, asked users to choose an exercise goal before building an exercise plan that included increased strength and power, building abdominal muscles, or controlling hypertension or diabetes, among others. In this app, if the user chose health-related exercise goals, the app asked the user to enter information related to their major CVD risk factors.

The fifth and sixth review criteria by which we evaluated the qualifying exercise apps was if the app provided FIT recommendations for the exercise plan for the four types of exercise. The ACSM introduced the importance of framing the ExRx by the FITT principle as the industry standard to establish the exercise dose that optimizes health [17,18,42,43]. Surprisingly, in this review, only ~5% of the exercise apps delivered their exercise programs by applying the entire FITT principle. Moreover, one third (~33%) of the exercise apps did not provide any information about the FIT for each type of exercise (Table 1 Question 5 and 6), but rather provided visual demonstrations of how the exercises should be performed.

The seventh review criterion by which we evaluated the qualifying exercise apps was if the app provided information on special considerations. Special exercise considerations are designed to optimize the health outcomes and reduce the risks resulting from exercise by adjusting the exercise plan for limitations imposed by past injury or the presence of chronic diseases and health conditions such as osteoarthritis and medication side effects, among others [17,24,30,44]. None of the 223 apps that focused on fitness provided special considerations along with the exercise plan (Table 1 Question 7). Surprisingly, only three of the apps of the nine that focused on health improvement for CVD risk factors and other chronic diseases and health outcomes provided special considerations as part of the exercise plan.

Our systematic review is the first to evaluate if any publicly available exercise apps met the professional standards of ExRx as specified by the ACSM [24,26,30], the AHA [2,25,38,39], and others [5,6,9]. Meeting these professional standards ensures that FITT ExRx can be produced by clinicians in a timely and systematic manner to optimize health outcomes and minimize the risks associated with exercise [45]. However, our study is not without limitations. We only reviewed apps that had more than 1000 reviews and were rated with at least four out of five stars. Considering the size of the exercise app market, we restricted our inclusion criteria to prioritize the exercise apps included, i.e., they were of high quality and had public reach. Given the dynamic nature of the app market [15], the exercise apps that met our inclusion criteria for this review may become outdated and replaced by newer, more advanced software technologies that emerge on the market.

## 5. Conclusions

The purpose of our systematic review was to evaluate commercially available exercise apps to determine if evidence-based exercise apps exist on the market that clinicians can use to prescribe individualized ExRx for adults with CVD risk factors and other chronic diseases and health conditions. Unexpectedly, we did not find a single exercise app clinicians could use to generate FITT ExRx that met the evidence-based professional standards of ACSM, AHA, and other professional organizations [2,24]. Indeed, only 1 exercise app of the 232 qualifying apps provided scientific evidence to support the foundation and delivery of their exercise programs. We acknowledge that none of the exercise apps we reviewed were purposefully designed for clinical use. Regardless, two thirds (~67%) of the exercise apps we evaluated did not meet any of the seven review criteria, which is concerning as users may not have adequate health literacy to ensure safety and optimize the health and fitness benefits of the recommended exercise program delivered by the apps. Our findings clearly deliver the message to primary care and family medicine practitioners that exercise is beneficial for adults with CVD or CVD risk factors. Our findings also established that there is an urgent need for exercise apps that produce evidence-based FITT ExRx that are readily available for clinical use.

## Figures and Tables

**Table 1:** The seven questions used to evaluate the exercise apps along with descriptive characteristics of the answers to each question.

Questions and Answers	Number of Apps	Percentage of Apps	Notes
**Question 1: Did the app specify the scientific foundation upon which the app was based?**			
A. Scientific evidence or guidelines from professional organizations.	*n* = 1	0.4%	
B. Expert’ or professional’ opinion.	*n* = 10	4.3%	
C. Neither A nor B.	*n* = 221	95.3%	
**Question 2: Did the app include an exercise preparticipation health screening function for users?**			
A. The app included an exercise preparticipation health screening function.	*n* = 0	0%	
B. The app recommended the user consult with professionals about preparticipation health screening in the legal disclaimer.	*n* = 80	34.5%	
C. The app had no information on exercise preparticipation health screening.	*n* = 152	65.5%	
**Question 3: Did the app build a CVD risk factor profile for users?**			
A. The app collected a comprehensive CVD risk factor user profile.	*n* = 1	0.4%	
B. The app collected some information on the CVD risk factor profile, but it was not comprehensive.	*n* = 90	38.8%	Information was collected on: Age: *n* = 82, 35.3% BMI: *n* = 74, 31.9% Baseline activity: *n* = 40, 17.2%
C. The app did not collect any CVD risk factor profile information.	*n* = 142	61.2%	
**Question 4: Did the app’s exercise plan focus on one or more of the user’s chronic disease(s) and/or health condition(s)?**			
A. The exercise plan focused on one chronic disease or health condition based on the health information collected from the users.	*n* = 1	0.4%	
B. The exercise plan focused on a named disease or health condition regardless of the chronic disease or health conditions information collected from the users.	*n* = 8	3.4%	
C. The exercise plan focused on body image or athletic performance. *	*n* = 155	66.8%	Exercise plan focused on: Body image: *n* = 71, 45.8% Weight loss: *n* = 124, 80.0% Athletic performance: *n* = 44, 27.1% Injury recovery: *n* = 7, 4.5%
D. The exercise plan did not have a clear focus regardless of the information collected from the user, if any.	*n* = 69	29.7%	
**Question 5: Did the app provide recommendations for all four types of exercise (i.e., aerobic, resistance, neuromotor and flexibility)? ^#^**			
A. The app provided recommendations all four types of exercise.	*n* = 10	4.3%	
B. The app provided exercise recommendations for aerobic and/or resistance exercise only.	*n* = 173	70.7%	Exercise recommendations were provided for: Both aerobic and resistance exercise: *n* = 80, 46.2%. Only aerobic or only resistance exercise: *n* = 93, 53.8%
C. The app provided various options and let the users choose which type of exercise to perform.	*n* = 49	21.1%	
D. The app provided no information about exercise types.	*n* = 0	0%	
**Question 6: Did the app provide the FIT information for each type of exercise recommended? ^§^**			
A. The app provided a comprehensive FIT information for each type of exercise recommended.	*n* = 42	18.1%	
B. The app provided some but not all the FIT information for each type of exercise recommended.	*n* = 116	50.0%	
C. The app provided no FIT information for each type of exercise recommended.	*n* = 74	31.9%	
**Question 7: Did the exercise app provide special considerations to ensure safety and maximize the health benefits of exercise?**			
A. The app provided a comprehensive list of special considerations for CVD Risk Factor(s), Existing Disease(s), and/or Health Condition(s) for users.	*n* = 0	0%	
B. The app listed some but not all special considerations for CVD Risk Factor(s), Existing Disease(s), and/or Health Condition(s) for users.	*n* = 3	1.3%	
C. The app had no information regarding special considerations for CVD Risk Factor(s), Existing Disease(s), and/or Health Condition(s) for users	*n* = 229	98.7%	

Note: CVD=cardiovascular disease; FIT = frequency intensity and time. All percentages in this table are calculated based on 219 total apps. * One app may have more than one focus. ^#^ For the types of exercise recommended, we were not able to review nine apps because payment was required. ^§^ For the FIT information for each type of exercise recommended, we were not able to review three apps because payment was required.

## Data Availability

No new data were created or analyzed in this study. Data sharing is not applicable to this article.
